# Mapping the intellectual structure of microfinance and women's empowerment: A bibliometric analysis

**DOI:** 10.1016/j.heliyon.2024.e39563

**Published:** 2024-10-18

**Authors:** Jenny Maldonado-Castro, Rocío Gallego-Losada, Antonio Montero-Navarro

**Affiliations:** a Department of Financial Economy and Accountancy, Facultad de Ciencias de la Economía y de la Empresa, Universidad Rey Juan Carlos, Paseo de los Artilleros, s/n, 28032, Madrid, Spain; b Department of Business Economics (Adm., Dir. and Org.), Applied Economics II and Fundamentals of Economic Analysis, Facultad de Ciencias de la Economía y de la Empresa, Universidad Rey Juan Carlos, Paseo de los Artilleros, s/n, 28032, Madrid, Spain; c Department of Business Sciences, Facultad de Ciencias Empresariales, Universidad Técnica Estatal de Quevedo, Av. Walter Andrade, Km 1.5 Via Santo Domingo, 120301, Quevedo, Ecuador

**Keywords:** Microfinance institutions, Women's empowerment, Bibliometric analysis, Sustainable development goals

## Abstract

The Sustainable Development Goals (SDGs) emphasize promoting and protecting women's rights and privileges. As a result, an increasing body of academic literature describes the various actions taken by different stakeholders to empower women. Amongst the initiatives implemented in many nations, microfinance and financial inclusion programs play a major role. This article aims to present an overview and synthesis of the research on the notion and practices of microfinance and its influence on entrepreneur women's empowerment. In order to do so, this study uses bibliometric techniques over a selection of papers extracted from the Web of Science database, to disentangle the knowledge structure of this academic field. According to our findings, the study of microfinance and women's empowerment is growing, with 470 publications, 963 authors, 67 nations and 36 research areas. Research topics include poverty reduction, gender issues in entrepreneurship, microfinance and women empowerment, and women in microcredit. An additional bibliographic coupling analysis has revealed the hottest research topics, showing the main gaps in the literature that suggest potential directions for future research.

## 1 Introduction

Women are gradually waking up, understanding their potential, utilizing available resources and working effectively in fields that match their skills [1]. Women's training influences the renewal of their thinking, action on gender development and the reconfiguration of their professional orientation, as well as initiatives to boost their capacity, empower them and create opportunities to contribute to their development [2,3].

Women are gradually assuming more equal roles, leading critical social changes and economic development [4,5]. The importance of the role of women is contemplated within the Sustainable Development Goals (SDGs) of the United Nations (UN) since they focus, among other things, on promoting and protecting women's rights and privileges in society, as well as women's equality and empowerment, within an inclusive and sustainable development framework that ensures social justice through the eradication of all forms of discrimination against women [6].

Women's empowerment is an effective tool for development, which must be viewed from a holistic perspective encompassing social, political, economic and legal elements [7,8]. Empowerment has a normative purpose embedded in a process that gives women access to and control over resources (both material and intangible) and the ability to influence decisions that will help women achieve their economic and social goals, giving them opportunities to engage in productive activities. Consequently, this would allow women to make strategic decisions and transform them into positive outcomes for their lives [[9], [10], [11]].

If the capabilities of people with low incomes (especially women) increase, they could overcome their vulnerabilities and benefit from new economic opportunities [12]. As a result, women would be more empowered, and consequently better prepared to make decisions in their daily lives in a society free of gender barriers [13,14]. Therefore, in recent years, scholars have developed a large body of literature involving the alternatives that stakeholders could take to empower women [15,16]. Financial inclusion through microfinance programs [17,18].

Microfinance is the provision of financial services by organizations to low-income consumers. Microfinance institutions (MFIs) have expanded their target to include nearly anyone, even those who could previously access mainstream financial services [19]. Indeed, some MFIs provide financial services to clients with little or no income to support fair and sustainable socio-economic development [[20], [21], [22]]. Therefore, it has the potential to be a crucial and successful instrument in the fight against poverty among marginalized people in developing countries [23], so they have been frequently reckoned as powerful tools to accomplish development goals, like SGD#1 (no poverty), SDG#5 (gender equality) and SDG#10 (reduced inequalities).

There is considerable literature assessing whether MFIs meet their poverty alleviation objective. Some researchers have found evidence to support this claim [24]. However, some scholars have reached different conclusions, finding no effects of MFIs on poverty reduction [25] and even an increase in poverty [26]. On the other hand, the absence of economic prospects and access to resources, like loans for women, has resulted in a feminization of poverty [27]. Therefore, women's economic empowerment is a crucial development component, and microfinance institutions are becoming critical tools in this effort [28].

According to Ref. [29], there are differing views on the link between microfinance and women empowerment: though some studies support a mainly positive association between both variables, other authors question the real benefits for women involved in such programs, showing that the access to financial resources does not necessarily generate empowerment. Under the adequate conditions, microfinances do not just contribute to the economic empowerment of women (improving their status, overcoming exploitation, enabling them to influence decisions …), but can also generate “virtuous spirals”, fostering an increase in their well-being, social and political empowerment and the achievement of gender equality goals [29]. Therefore, promoting the potentially positive influences of microfinance on women empowerment requires addressing underlying gender norms, social structures, and power dynamics [29]. Following [29], services from green microfinance institutions help women when creating green start-ups, increasing their economic security and freedom.

Several scholars have focused their research on examining the effectiveness of microfinance programs [30,31]. As a result, there is an abundance of academic literature, which has led several authors to analyze this research stream through systematic literature reviews [32,33]. Furthermore, the body of literature has increased exponentially in recent years, so several researchers have used a bibliometric approach to study this vast literature [34]. Table 1 presents a brief summary of bibliometric articles dealing with microfinance.

**Table 1:** Literature review of bibliometric articles analysing microfinance.

No.	Reference	Time Span	Database	Theme	Number of documents	Keywords	Future research
1	[35]	1996–2019	Scopus	25 years of microfinance	1429	• Bibliometrics analysis • Citation analysis • Literature review • Most cited documents • Three-field plot • Trend topic • Word cloud	• Comparative research on loan lending models and governance • Pricing models and strategies in MFIs • The role of Islamic Microfinance in supporting sustainability and addressing social and environmental issues • Comparing MFI performance with other financial institutions
2	[36]	1996–2022	Scopus and WOS	Gender diversity and its impact on the performance of MFIs	24	• Gender diversity • Financial performance • Social performance • Social outreach • Microfinance institutions	• Impact of gender diversity on borrowers • Impact of gender diversity on the efficiency and productivity measures of MFIs • Potential nonlinear or quadratic relationships, identifying optimal thresholds for gender diversity impact on MFIs performance
3	[37]	2006–2023	Scopus	Impact of financial inclusion on investment decisions	161	• Financial inclusion • Investment decision • Bibliometric analysis • Research trends	• Financial inclusion and development • Attitudes and expertise in Economic Growth • Fintech and Social Impact
4	[38]	1979–2023	Scopus	Integration of Islamic social and commercial finance	632	• Integrations of Islamic social and commercial finance • Systematic literature review • Integration model • Integration behaviour • Integration benefits and impact • Integration issues and challenges	• Integration of Islamic Social finance with commercial institutions. • Influence of Islamic MFI son the muslim community. • Social-commercial financial engineering and its consistency with Islamic principles • Policy and legal framework required to obtain a comprehensive analysis of the integration of Islamic social Finance and commercial institutions.
5	[39]	2017–2022	Scopus	Microfinance for SMEs	388	• Microfinance • Microfinance institutions • Small and medium enterprises (SMEs) • Microcredit • Bibliometric analysis	• Sustainability of informal sources of credit and their impact on SMEs performance. • Financing models and patterns of MFIs • Sustainability of Islamic finance • Crowdfunding in developing countries • Regulatory and policy frameworks for MFIs
6	[40]	1998–2021	Web of Science	Microfinance and Information and communication technologies (ICTs)	347	• Microfinance • Microcredit • Microbanking • Microsaving • Microlending • Microinsurance • ICT	• The contribution of fintech to financial inclusion and empirical evidence-based impact measurement. • How managers or investors react to technological developments and how they affect Microfinance Providers' (MFP) operations. • The control of the risks associated with these new technologies. • Empirical studies look at whether and how financial inclusion affects entrepreneurship.
7	[41]	2012–2021	Web of Science	Trends in Microfinance outcomes	524	• Microfinance • Microcredit • Microbank • Microfinance institution • Performance • Success • Outreach • Impact	• Future research should avoid politics and consider the macro context rather than relying on limited testing. • Investigations into crowdsourcing. • Research that investigates soft information with innovative selection and social credit models. • A more comprehensive strategy is needed in microfinance research to combat poverty and advance global economic and human development.
8	[42]	1989–2019	Scopus	Trends on microfinance institutions and microfinance	4681	• Microfinance • Microcredit • Microbank • Microinsurance • Microsaving • Microfinance institution • Financial exclusion	• Investigations that deepen the conceptual framework to link the abundance of natural resources, financial development and poverty reduction that lead to economic growth. • Formulation of research theories and border methods for the suggested conceptual framework. • Formulation of advanced economic methods and blockchain technology based on the conceptual framework. • Design and coding of application software for banking to expand the financial inclusion base and scope development. • Studies investigating the role of income/income of natural resources, financial development and their functions in reducing poverty that leads to economic growth.
9	[43]	1998–2021	Scopus	Microfinance Institutions and women empowerment	395	• Microfinance institutions • Women empowerment	• Does not provide information.
10	[44]	2000–2020	Scopus	Islamic Microfinance	122	• Microfinance • Microcredit • Small medium enterprise • Islam • Sharia	• Future research that evaluates the achievements of the Islamic Microfinance Institution (IMFI) in the light of the principles Maqasid al-Shari'ah. • Studies that explore the specific challenges of each country for the sustainability of the IMFI. • Comparative studies between conventional and Islamic IMFs within a region or country. • Future research that tests the practical application of these business models. • Future studies should explore a different aspect of mitigating poverty through Islamic microfinance. • Research that explores the role of Islamic microfinance to guarantee low-cost homes, water supply, sanitation, and health services. • Studies that address the initiatives adopted by Islamic microfinance to protect the environment. • Future research on the financial decision-making of microentrepreneurs. • Increase empirical research that explores the way IMFIs help the empowerment of women. • Future research should explore how Shari'ah aspects of microfinance products affect Islamic banks' current supply of products. • Explore evidence on the impact of good governance on the results of the IMFI. • Explore the role of members of the Shari'ah council in Islamic microfinance institutions. • Present in-depth market studies to better learn the market size and customers' needs.\
11	[45]	1995–2020	Scopus	Microfinance performance	1252	• Microfinance • Microcredit • Microlending • Microbank • Performance • Outreach • Efficiency • Productivity • Trade off • Social performance • Financial performance	• Future research focused on the performance of the MFIs as a financial institution from a border perspective. • Comparative studies of the MFIs performance with other financial institutions. • Future research focused on aspects of institutional efficiency and financial stability.
12	[46]	2007–2021	Scopus	Islamic Microfinance in Islamic economic and financial research	208	• Islamic Microfinance	• New research about Islamic microfinance with a broader theme. • Improve research on Islamic microfinance, especially in Indonesia.
13	[47]	1993–2019	Web of Science	Microfinance	2168	• Microfinance	• Future studies on the role of MFIs in the financing of the seed phase of new projects and companies, especially in developed countries.
14	[48]	1997–2017	Web of Science	Microfinance	1874	• Microfinance • Microcredit • Microbank • Microsavings • Microinsurance	• The latest shocking subjects are financial inclusion, social entrepreneurship, and Islamic microfinance. • Research that not only measures the impact of microcredit but also identifies the conditions in which it works better.

As seen in Table 1 [43], published a bibliometric article on Microfinance Institutions and Women Empowerment using the Scopus database, using the keywords “Microfinance Institutions” AND “Women empowerment”, which may leave some relevant articles out of the selection, especially some ones related with social development. The present article follows a systematic approach using the Web of Science database, one of the most popular ones amongst the academic community [49]. Furthermore, in order to select the most relevant articles, the search strategy is expanded. In addition, this article delves into the explanation of the results, specifying the hottest trends and future research lines, filling a gap in the academic literature.

Therefore, this article aims to provide an overview and synthesis of research on the notion and practices of Microfinance and its influence on women's empowerment through a bibliometric approach that enables the assessment of its cognitive structure, evolution, and trends in this field of study. Bibliometric analysis is widely spread as a means to disentangle the intellectual structure of an academic field, including many tools and techniques which range from productivity analysis to mapping instruments [50,51]. This general objective is broken down into the following specific objectives (1) to assess academic productivity through the historical evolution of publications; (2) to determine the intellectual structure of the research topic; (3) to discover the thematic organization; (4) to analyze specific research trends related to the microfinance industry and women's empowerment. According to these objectives and following recent bibliometric reviews, some research questions can be stated.

RQ1 What is the historical evolution of the literature on microfinance institutions (MFIs) and women's empowerment research?

RQ2 What are the most prominent papers that have influenced the intellectual structure of microfinance and women's empowerment research?

RQ3 Who are the most productive authors, countries, and journals around which microfinance and women's empowerment research is organized?

RQ4 What are the main topics in microfinance and women's empowerment research?

RQ5 What are the hottest topics and research trends in microfinance and women's empowerment research?

This article provides scholars with an overview of the current status and trends in this body of research. The results of this study are particularly relevant given the need to move towards women's equality and empowerment within a socially and economically inclusive and sustainable development framework.

In terms of structure, the paper begins with an overview of the study of microfinance and women's empowerment, followed by a description of the data collection and analysis techniques used in this research. The following section provides information on the results, delving deeper into microfinance and women's empowerment through performance analysis and science mapping. Finally, the article presents its key findings and reflections through discussion, conclusions, limitations, and future research directions.

## 2 Methodology

The evaluation of scientific literature requires the use of analytical review strategies [52]. Systematic reviews are crucial to broadening our understanding, consolidating information and identifying new research directions [53,54]. Bibliometric tools have become great allies of researchers to explore the literature of various academic fields, since they allow the study of scientific production and its returns in terms of authors, countries, institutions, and journals [[55], [56], [57]].

**Fig. 1:**
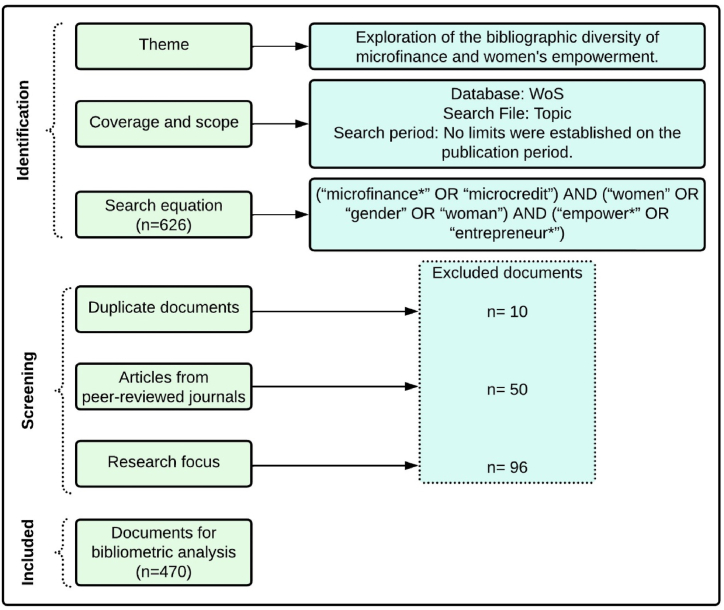
Selection process using PRISMA methodology.

These reviews require a rigorous process which will avoid potential errors when conducting an extensive literature review [58,59]. Using the Preferred Reporting Items for Systematic Reviews and Meta-Analyses (PRISMA) framework grants the adequate levels of rigor and transparency along the entire process [60]. Fig. 1 shows the selection criteria used following PRISMA methodology.

The information search strategy was applied on the Web of Science (WoS) bibliographic database, one of the most relevant in social sciences, frequently used for bibliometric studies in business and economics. This bibliometric data source is frequently used in SDG-related literature [61]. Furthermore, WoS indexes fewer journals than the popular Scopus due to its strict indexing, allowing for a broader range of high-quality journals [62]. In order to avoid the subjectivity of researchers in data collection, the authors applied a keyword search. The search string is: (“microfinance∗” OR “microcredit”) AND (“women” OR “gender” OR “woman”) AND (“empower∗” OR “entrepreneur∗”).

This search followed several criteria for the selection of documents: (i) including only articles from peer-reviewed journals, which ensures the quality of their content [63]; (ii) the language chosen was English, the predominant language used by the academic community [64]; and (iii) no limits were set on the publication period to cover as many publications as possible until the end of 2021. In order to carry out an additional bibliographic coupling analysis, the same search strategy was used considering the period 1/1/2022-15/4/2024. As a result, the total number of documents obtained was 626.

Over this first selection of papers, the authors removed duplicates and analyzed their content to confirm their fit with the object of study. After this filtering, the final selection includes 470 articles.

The analytical tools provided by WoS allowed the obtention of the measures of productivity, considering the historical evolution of the publications (RQ1), the most influential ones (RQ2) and the leading journals (RQ3). The VOSviewer software allowed, in turn, science mapping, which researchers from various academic disciplines widely use due to its suitability for presenting bibliometric maps that facilitate the interpretation of their cognitive structures [65,66]. In this case, we have used VOSviewer to determine the evolution of the intellectual structure and the hottest research trends related with the knowledge field of women's empowerment and microfinance (RQ4 and RQ5).

## 3 Results

### 3.1 Performance analysis

#### 3.1.1 Productivity

**Fig. 2:**
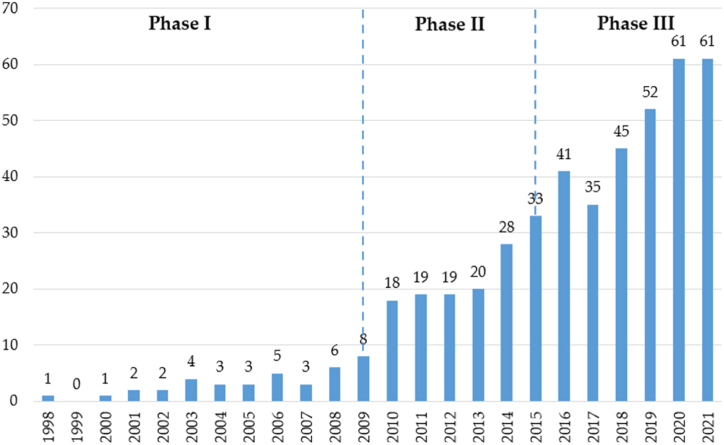
Historical evolution of publications (1998–2021).

Fig. 2 presents the evolution of the scientific production of 24 years of research on the practices of Microfinance and its relationship with women's empowerment. During this period, the academic community has published 470 scientific articles. In the first years, studies on this topic were scarce. However, there is a growing interest in the academic community, concentrating 54 % of the scientific production in the last five years (2017–2021).

The first publication, in 1998, was [67]. The authors presented a paper investigating the connection between poor women's participation in microcredit programs and their empowerment through a sample of empirical data from rural Bangladesh.

Considering the evolution of the number of publications, we can divide the scientific production in this field into three main periods, which we will refer to as pioneering works (1998–2009), laying the foundations (2010–2015), and scientific consolidation and development (2016 onwards).

In the first period (1998–2009, pioneering works), 8 % of the scientific output was published (38 papers). These articles focus on the study of microcredit as a strategy that governments have applied to combat poverty and empower minorities, with a particular focus on women [[68], [69], [70]]. Furthermore, at this stage, the first research on women entrepreneurs [71] and their financial practices [72] began to be addressed, paying particular attention to the challenge of access to credit [73] and repayment [74].

From 2010 to 2015 (laying the foundations), the researchers published 29 % of the scientific production (137 articles), including the article with the highest impact in the selection, with 449 citations [75]. At this stage, researchers address the impact of access to financial services on the economy of women and their households [76,77]. In addition, the debate about validating the argument that microcredit reduces poverty is opened [78,79].

From 2016 to 2021 (consolidation and scientific development), there was a significant increase in scientific production, including 295 articles (63 %). At this stage, the debate about the role of microfinance in poverty reduction continues. For example [80], found that microfinance exacerbated economic, social and environmental vulnerabilities, even increasing debt levels among people who were already vulnerable [81]. argue that, while microcredit can be beneficial, its impact on the disadvantaged is increased and sustained if the dangers of the poverty trap are addressed with microinsurance; therefore, combining microcredit with microinsurance will enable the disfavored to lift themselves out of poverty in the long run. Meanwhile, research findings from various scholars reveal that microfinance interventions prioritizing loan availability and social mediation raise the living standards of women entrepreneurs and their families [82,83].

Similarly, in this period, some authors investigated the impact of microfinance participation on women's economic, social, psychological, and political empowerment. Some papers found positive effects on women's empowerment, due to their access to financial services [84,85]. However, other authors found that the effectiveness of this practice yields contradictory results [86,87].

Poor women are particularly vulnerable to domestic violence [88,89], so in order to increase women's social status and decrease the gender gap, economic empowerment is a critical part of social policy formulation [90]. This perspective has caught the attention of several scholars during this period, publishing studies to examine the relationship between domestic violence and participation in microfinance programs [91,92]. [93] recommend researchers to distinguish between women borrowers who have control over their credit and those who do not since, the decrease in physical violence against women who manage credit is notable. Indeed, participating in microfinance programs can improve the likelihood that women will join together to fight domestic abuse and spousal abandonment [94]. However, evidence supports the possibility of spousal backlash, perhaps due to a struggle for dominance over household resources [91,95].

Finally, the COVID-19 pandemic conditioned the research agenda. For example [96], argue that the promotion of microfinance as a market-based means of pandemic relief and recovery should raise the alarm rather than comfort for three reasons: it increases the use of credit; reliance on MFIs can leave households undernourished; and it poses a significant threat to gender equality and sustainable development [97]. focused their study on examining, from a qualitative point of view, how COVID-19 confinement has affected women-led microenterprises that have obtained loans from MFIs, revealing the need to take gender gap issues into account when designing COVID-19 response policies in developing countries. Meanwhile [98], demonstrated how specific improvements developed by MFIs during the COVID-19 pandemic promote further consolidation of NGOs in Bangladesh society.

#### 3.1.2 Most productive countries

**Fig. 3:**
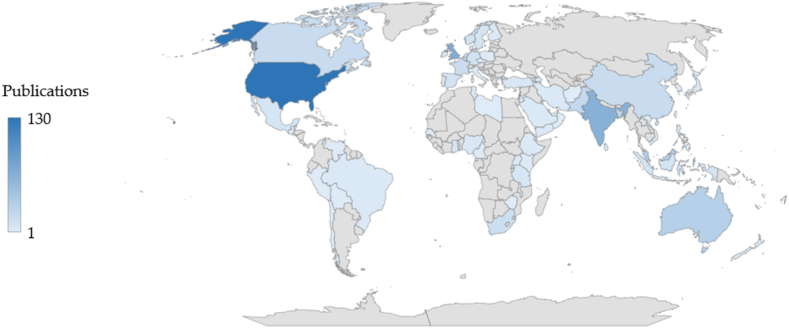
Countries' contribution (1998–2021).

The analysis of the most productive countries is carried out based on the author's affiliation, allowing us to know the various existing relationships between countries to generate knowledge [99]. Fig. 3 shows the participation of researchers belonging to 67 countries, with the top 10 including five Asian countries (India, Malaysia, Bangladesh, Pakistan, and China), two European countries (United Kingdom and the Netherlands), two from the Americas (United States and Canada) and one from Oceania (Australia).

The United States, the United Kingdom, Canada, and Australia are just a few of the developed nations in this list of the top 10, because they adhered to international organizations’ rules, including those of the World Bank, and their support for microfinance [[100], [101], [102], [103]].

Similarly, several developing nations, including China, India, Bangladesh, Malaysia, and Pakistan, have adopted microfinance programs. Initiatives involving Self-Help Groups (SHGs) predominate in Bangladesh and India. In fact, microfinance institutions started to appear when economist Muhammad Yunus established The Grameen Bank in 1983 as the first bank that only provided small loans to poor women in rural Bangladesh [104]. In Malaysia, the well-known Amanah Ikhtiar Malaysia (AIM) program has been running since 1987 and offers loans to underprivileged rural residents [105]. The Rural Credit Cooperative (RCC) microcredit program, which is China's primary source of microfinance, was established by the Government in 2000 [106]. According to the State Bank of Pakistan [107], Pakistan joined the microfinance business later, through Microfinance Banks (MFBs), in 2001. Nonetheless, the reach of MFBs has significantly risen [108]. As a result, these programs have assisted the most disadvantaged, empowering them [85,[109], [110], [111], [112]].

On the other hand, the Commonwealth appears to have had a relevant impact, since seven of its members—India, Malaysia, Bangladesh, Pakistan, United Kingdom, Canada, and Australia—appear in the top 10. The Commonwealth has been a strong proponent of gender equality through its Declaration on Gender Equality and Plan of Action on Gender Equality and Women's Empowerment, which acknowledge gender equality as a tool to end poverty, spur economic progress, and foster sustainable and peaceful development. Consequently, more gender equality in the workplace and school leads to a more productive workforce, encouraging increased investment and growth [113].

The United States is the most productive country, with 130 publications. Authors such as Nadine Shaanta Murshid (8 publications), Erin Beck (3) and Garry Bruton (3) stand out. Among the main topics addressed by authors from this country are the exploration of the role of Self-Help Groups (SHGs) in development [114,115] and women's empowerment [84,116,117]; as well as women's entrepreneurship [73,118]. It is worth mentioning that SHG programs include small groups of volunteers who share an affinity and help each other, helping women to access working capital [119].

Similarly, US researchers have collaborated with colleagues from 52 countries, especially Bangladesh, with whom they have six joint publications. These articles study microcredit programs for women in Bangladesh, specifically BRAC (Bangladesh Rural Advancement Committee), because of its prominent role in women's empowerment, which has been one of the country's main objectives for national development [120,121]. Similarly, other researchers analyze the impact of these programs on different areas of beneficiaries' lives [67,[122], [123], [124]].

The second most productive country is the UK, with 69 publications. The studies with the most significant impact produced in this country address various topics, such as the comparison between the evaluations carried out by other authors on the impact of credit on female borrowers [68], the extent of women's empowerment in reducing intimate partner violence [69]; and the analysis of the effect of patriarchal control of assets in the study of female empowerment through access to credit [70]. Remarkable authors include Supriya Garikipati and Samia Mahmood, who have published five papers each.

UK researchers have collaborated with academics from 42 nations, with South Africa being their most prominent partner, cooperating in seven papers. These studies focus on systematic reviews, analyzing how financial growth has helped low-income people access human development opportunities [125], as well as the influence of microfinance on the lives of poor women, men and children in sub-Saharan Africa, particularly on longer-term non-financial outcomes related to education, health and nutrition [126]. Similarly, the remaining publications explore the fruits of the IMAGE (Intervention with Microfinance for AIDS and Gender Equity) program, run by the Small Enterprise Foundation in South Africa, aiming to facilitate women's access to credit and provide group support. It is worth noting that according to the findings of various researchers, this initiative has favorable results in terms of fostering specific indicators of empowerment [69,127,128], couple relationships [129], as well as improvements in the community [130].

India is the third most productive country, with 64 publications. The studies with the most significant impact in the academic community produced in this country address various topics, such as the differences between the impact of financial inclusion programs on impoverished households represented by women and men [131], the complexity and diversity of women's informal financial activities [72]; and the behavior of husbands and wives regarding the use of health insurance included in microcredits [132]. In addition, the author Isabelle Guérin, who has four articles published with the affiliation of French Institute Pondicherry in India, stands out.

Twenty-three countries, being the United States the outstanding partner, have collaborated in the India-led research, with five published studies. These articles focus on issues such as evaluating the investment effectiveness of financial inclusion programs [117,132,133], specifically on self-help programs targeting women [115,116].

#### 3.1.3 Most productive journals and research areas

As shown in Table 2, the spread the research on microfinance practices and their influence on women's empowerment has been published in 269 journals. World Development leads the scientific production, including 28 articles. This journal publishes studies that explore ways to improve living conditions by disseminating solutions to different problems, including gender discrimination and lack of popular participation. The highest impact article (438 citations) published in this journal is authored by Naila Kabeer, who investigates the factors that have led to divergent conclusions about the ability of credit programs to empower rural women in Bangladesh. According to her findings, the leading cause of the conflict is the highly diverse understandings of intra-household power relations based on these studies, even though these evaluations use relatively different methodologies and are conducted at different points in time [68].

**Table 2:** Distribution of articles by most influential journals and research areas.

R	Journal	N	%	HI	SJR	Research Areas	N	%
1	World Development	28	5.96	192	2.3	Business Economics	212	45.11
2	Journal of Development Studies	13	2.77	93	0.95	Development Studies	115	24.47
3	Development in Practice	11	2.34	45	0.47	Women S Studies	38	8.09
4	Development and Change	9	1.91	96	1.61	Social Sciences Other Topics	35	7.45
5	International Journal of Gender and Entrepreneurship	8	1.70	33	0.67	Social Work	24	5.11
6	Journal of International Development	7	1.49	69	0.6	Public Environmental Occupational Health	19	4.04
7	Pacific Business Review International	7	1.49			Anthropology	15	3.19
8	Journal of Interpersonal Violence	6	1.28	112	0.91	Sociology	15	3.19
9	Gender in Management	5	1.06	54	0.78	Government Law	14	2.98
10	Journal of Business Ethics	5	1.06	208	2.44			

R = Rank; N = Number of Publications; % = Percentage of total publications; HI = H-Index; SJR = SCImago Journal Rank 2021.

Table 2 also shows that there are journals that analyze microfinance and women's empowerment from different points of view: Business Economics, related to aspects of the economic sphere, as well as financial and business elements that involve theory and applications at the micro and macroeconomic level; Development, which seeks to understand the social, economic and cultural aspects of society; and Women's Studies, aimed at disseminating studies on the social condition of women.

#### 3.1.4 Featured authors

The author's analysis provides information on the scholars who have generated knowledge in this field [66]. The 470 articles have been written by 963 authors. Table 3 shows the most prolific authors, who have published at least four articles on this topic. The participation of Nadine Shaanta Murshid, Isabelle Guérin, and Abdullah Al Mamun, who led the table by publishing 8, 7, and 6 articles, respectively, stands out.

**Table 3:** Most prolific authors in Microfinance and Women Empowerment.

R	Author	Country	University/Institution	N	H-Index
1	Murshid, Nadine Shaanta	United States	State University of New York (SUNY) System	8	9
2	Guérin, Isabelle	India	French Inst Pondicherry	7	14
3	Mamun, Abdullah Al	Malaysia	Universiti Kebangsaan Malaysia	6	15
4	Garikipati, Supriya	United Kingdom	University of Liverpool	5	9
5	Mahmood, Samia	United Kingdom	University of Wolverhampton	5	5
6	Rashid, Nurulizwa	Malaysia	Tech Univ Malaysia Malacca	5	5
7	Szafarz, Ariane	Belgium	Université Libre de Bruxelles	5	17
8	Agier, Isabelle	France	Universite Paris Cite	4	9
9	Hansen, Nina	Netherlands	University of Groningen	4	14
10	Lensink, Robert	Netherlands	Wageningen University & Research	4	29

R= Rank; N = Number of Publications.

Nadine Shaanta Murshid is a researcher whose publications have focused on exploring microfinance and its potential for positive outcomes for the women who participate in it, looking at control of resources [134,135] and justification of partner violence [134,136]; however, she found no results to support the positive effect of microfinance on women's empowerment. In addition, one of her papers focuses on the effect of Microfinance Institutions (MFIs) innovations during the COVID-19 pandemic on NGOs in Bangladesh [98].

Similarly, Isabelle Guérin conducts her research with a gender approach. Her publications focus on the impact of microcredit on the achievement of gender equality [86], studying its impact on women's empowerment [137] and the costs involved in achieving this empowerment [138]. As a result, the author criticizes how these business practices are carried out [72,139] by demonstrating evidence of unexpected effects [140,141].

Finally, Abdullah Al Mamun focuses his studies on women-led microenterprises. Therefore, the author analyzes entrepreneurial traits involving development initiatives [142], performance [143,144], sustainability of enterprises [145]; as well as the impact of microcredit on socio-economic development [146].

#### 3.1.5 Most-cited publications

A widespread technique to examine the performance of an academic field is the analysis of the number of citations received by the publications [147]. Table 4 shows the articles with the highest number of citations in absolute terms ordered by citations per year (C/Y). These publications study the impact of microfinance programs in different areas, such as women's empowerment [68,69,148], reduction of intimate partner violence [69], socio-economic outcomes [75,76,78], poverty reduction [79,80] or business performance and governance [149]. In addition, one of these studies examines the profiles and characteristics of borrowers who engage in online microfinance activities [150].

**Table 4:** Most frequently cited publications in Microfinance and Women empowerment.

R	Title	Authors	Year	Journal	TC	(C/Y)
1	The Miracle of Microfinance? Evidence from a Randomized Evaluation	Banerjee, Duflo, Glennerster & Kinnan	2015	American Economic Journal-Applied Economics	449	64.1
2	Understanding the impact of a microfinance-based intervention on women's empowerment and the reduction of intimate partner violence in South Africa	Kim, Watts, Hargreaves, Ndhlovu, Phetla, Morison, Busza, Porter & Pronyk	2007	American Journal of Public Health	336	22.4
3	Conflicts over credit: Re-evaluating the empowerment potential of loans to women in rural Bangladesh	Kabeer	2001	World Development	438	20.9
4	The Impacts of Microfinance: Evidence from Joint-Liability Lending in Mongolia	Attanasio, Augsburg, De Haas, Fitzsimons & Harmgart	2015	American Economic Journal-Applied Economics	134	19.1
5	Microfinance and the business of poverty reduction: Critical perspectives from rural Bangladesh	S.B. Banerjee & Jackson	2017	Human Relations	79	15.8
6	Microcredit in Theory and Practice: Using Randomized Credit Scoring for Impact Evaluation	Karlan & Zinman	2011	Science	170	15.5
7	Female leadership, performance, and governance in microfinance institutions	Strom, D'Espallier & Mersland	2014	Journal of Banking & Finance	122	15.3
8	Microcredit: Empowerment and Disempowerment of Rural Women in Ghana	Ganle, Afriyie & Segbefia	2015	World Development	105	15.0
9	Microfinance Decision Making: A Field Study of Prosocial Lending	Galak, Small & Stephen	2011	Journal Of Marketing Research	160	14.5
10	The Impacts of Microcredit: Evidence from Ethiopia	Tarozzi, Desai & Johnson	2015	American Economic Journal-Applied Economics	99	14.1

TC = Total Citations; C/Y = Number of citations per year.

The most cited paper in relative (64 C/Y) and absolute terms (449 citations) is mentioned above, published by Banerjee, Duflo, Glennerster and Kinnan in 2015. In this paper, the authors present a randomized evaluation of a cluster microcredit program in India, finding an increase in the investment in small businesses and the profits of incumbent firms. However, the scholars found no significant changes in women's health, education or empowerment [75].

### 3.2 Science mapping

#### 3.2.1 Co-occurrence cited authors network

**Fig. 4:**
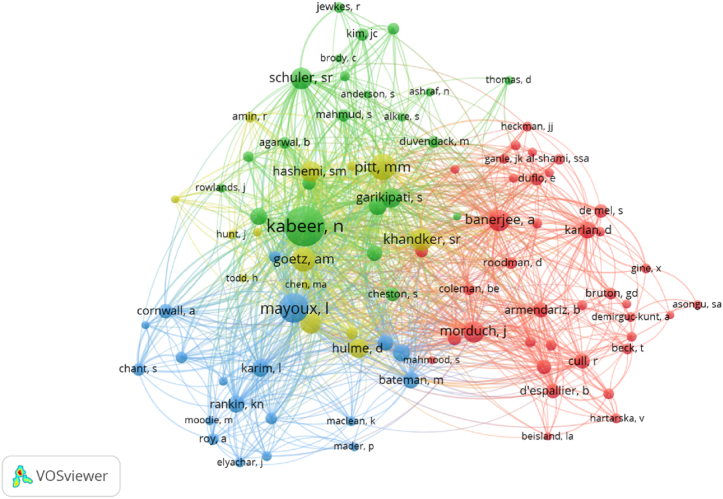
Co-occurrence network of cited authors in the field of Microfinance and Women Empowerment (1998–2021).

VOSviewer was used to visualize the co-occurrence network of authors shaping the intellectual structure of the academic field of Microfinance and Women Empowerment. The database contains 11,564 cited authors; however, only 94 have, at least, 20 citations. Fig. 4 presents the co-occurrence network of cited authors, gathered in four clusters.

Cluster 1 (red color), 'Poverty alleviation', comprises 38 nodes with 1531 citations. In this cluster, prominent authors A. Banerjee, E. Duflo and D. Karlan have shared publications to examine in depth the effectiveness of microcredit as a development tool [151,152]. In recent years, different financial aid initiatives have been enacted, aiming at enabling participants to achieve a sustainable standard of living and, thereby, alleviate poverty [75,153]. Morduch is also a prominent author who has conducted several pieces of research on the potential of microfinance to alleviate poverty by providing credit to poor households in order to spread financial services [[154], [155], [156]]. Finally, it is worth noting that Morduch and Armendariz, also included in this cluster, jointly published a book analyzing the global expansion of financial markets in poor communities [157].

Cluster 2 (green colour), 'Credit as a tool for empowerment', comprises 23 nodes with 1459 citations. The most prominent author in this cluster is Kabeer (436 citations). She is a well-known academic researcher due to her extensive contribution to the literature on empowerment processes [158]. In her articles, she seeks to analyze methodologies for measuring women's empowerment [9,68]. Similarly, Garikipati has followed this research line studying the impact of loans on women's empowerment. Her findings in India showed that female workers who have access to productive resources are significantly increasing their agency in both the family and the labor market [159]; however, the author has shown that the money obtained from loans is sometimes diverted to improve household income, thus calling for the need to ensure women's control over the assets created by loans [70,160]. Meanwhile, Swain focuses on the role of SHGs as a source of finance and their effect on female empowerment [[161], [162], [163], [164]]. Similarly, Schuler has studied the effect of women's participation in rural credit programs on their empowerment, finding favorable results in Bangladesh [165,166].

Cluster 3 (blue color), ‘Financial Inclusion Policies’, comprises 20 nodes with 1057 citations. Mayoux outstands for her studies on gender issues involving women's inequality and participation in financial inclusion programs [167]. She highlights the need for more local participatory projects and broader movements for change in the development agenda [168]. Meanwhile, Karim, in her publications, examines the role of gender in the growth of globalization and neoliberalism in Bangladesh [169], which is intensified by the implementation of microfinance programs targeting poor women [170]. Rankin reflects on the gender-sensitive design of microfinance programs in development policy-making [171,172]. Finally, Guerin focuses her studies on exploring the ambiguities in financial inclusion initiatives and highlights the urgency of restructuring these policies [72,173].

Cluster 4 (yellow color), ‘Microcredit Outcomes’, comprises 21 nodes with 1013 citations. This cluster includes prominent authors such as Pitt and Khandker, who have jointly published papers focusing on exploring the effects of credit programmes on individual and household welfare, considering different aspects such as education, labour supply health [174,175] and empowerment [176]. Meanwhile, Hashemi focuses on men's violence towards women and how it can be fought through women's economic and social independence achieved with their access to credit [[177], [178], [179]]. Furthermore, Hulme reviewed the methodological options to evaluate the effects of microfinance programs on development initiatives [180]. He also assesses the veracity of the premise that poverty could be reduced through lending to microentrepreneurs. According to his results, most of the programs examined had favorable effects on poverty and income, but only modest positive effects on employment and technology [181]. Similarly, Rahman explores the theory which proposes that microcredit programs have the potential to promote equitable and sustainable development. His results show that they could increase violence in society and produce new forms of domination over women [21].

#### 3.2.2 Co-occurrence keyword network

**Fig. 5:**
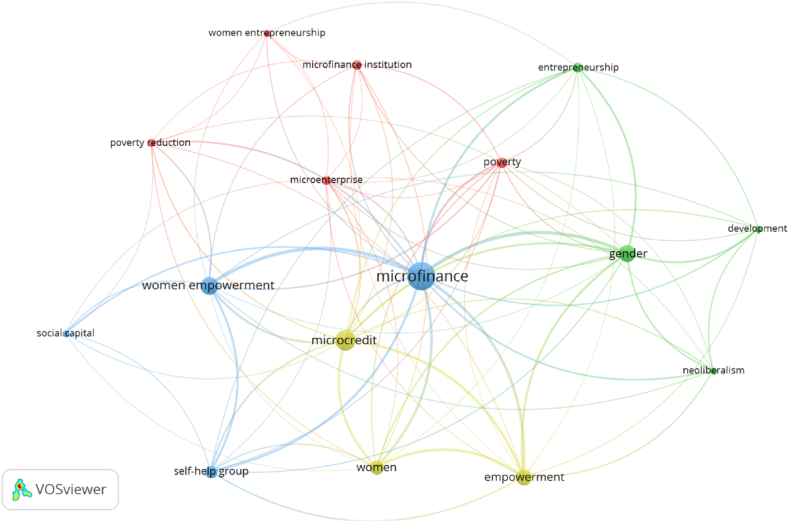
Co-occurrence network of author's keywords in the field of Microfinance and Women Empowerment (1998–2021).

In order to represent the intellectual structure of the academic field of Microfinance and Women Empowerment and its most relevant topics. VOSviewer software has been used, to visualize the keywords proposed by the authors of the different papers. The database contains 803 keywords; however, only 16 co-occur at least 10 times. Fig. 5 presents the co-occurrence network of the author'’ keywords, including 38 nodes (relevant topics) and four clusters (research themes).

Cluster 1 (red), ‘Poverty reduction’, comprises five nodes with 88 occurrences. The themes of these papers focus on poverty reduction, whereby various authors discuss the roles of microenterprises, microfinance organizations and women entrepreneurs. Several authors included in this cluster examine policymakers' attempts to fight poverty using such tools [80,182], with financial inclusion programs being examined [[183], [184], [185]]. As a result, researchers have analyzed their outcomes [[183], [184], [185]]. Several academics have also discussed entrepreneurs' critical role in reducing poverty [186,187], even focusing specifically on women microentrepreneurs and the financial difficulties that their businesses face [[188], [189], [190]].

Cluster 2 (green color), ‘Gender issues in entrepreneurship’, comprises four nodes with 106 occurrences. The topics addressed by these papers include gender, entrepreneurship, development and neoliberalism. For example, several authors focus their studies on gender issues in entrepreneurship [191], such as gender equity in microfinance [192], financing female entrepreneurship [193] and gender bias in loan access [188,[194], [195], [196]]. Additionally, some academics have focused their studies on the political economy of these programs, since the neoliberal ideals of the individual entrepreneurial initiative appear to fit with the kind of development aid that microcredit offers [197,198].

Cluster 3 (blue color), ‘Microfinance and Women Empowerment’, comprises four nodes with 276 occurrences. As seen in Fig. 5, the ‘microfinance’ node is in the middle of the network, indicating its prominence and closeness to the other nodes. These cluster's themes are microfinance, women's empowerment, self-help groups and social capital. Some authors examine the role of microfinance programs in development strategies [180,183,199] and how they affect many aspects of the economy, society, and health [130,161,200], including the improvement of social capital in poor communities [80]. Similarly, other academics analyze how microfinance contributes to women's empowerment [161,183,201]. Furthermore, some researchers evaluate the impact of self-help groups (SHGs) on women's lives, including the process of empowerment [84,202,203], lifestyle modifications [204], involvement in political and social activities [205,206], and economic development [207].

Cluster 4 (yellow color), ‘Women in microcredit’, comprises three nodes with 181 occurrences. The themes of this cluster are microcredit, empowerment, and women. Several academics focus on examining how women use microcredit. For instance, some papers have studied how loan availability affects the financial status and health of female borrowers and their households [208,209] and more fundamental issues like female empowerment [85,148,210,211]. Similarly, several studies focus on the challenges women face when acquiring credit [31,212], how they employ valuable microcredit resources [213], and how this affects their ability to act as entrepreneurs [214,215]. Finally, some authors study women's challenges when repaying loan debt [71,216].

#### 3.2.3 Bibliographic coupling analysis

In order to determine the hottest trends inside a research field, bibliographic coupling is placed amongst the most popular bibliometric techniques [217]. Coupling happens when two articles cite the same reference, which reveals a high probability of relationship between them, as long as they are based on the same empirical or theoretical bases [51]. Bibliographic coupling could act as an ideal complement of co-word analysis, as it delivers a better treatment of the most recent literature, being used by most of the literature in order to analyze specifically the most recent papers. As a result, coupling has been frequently used by the finance academic community [218,219].

**Fig. 6:**
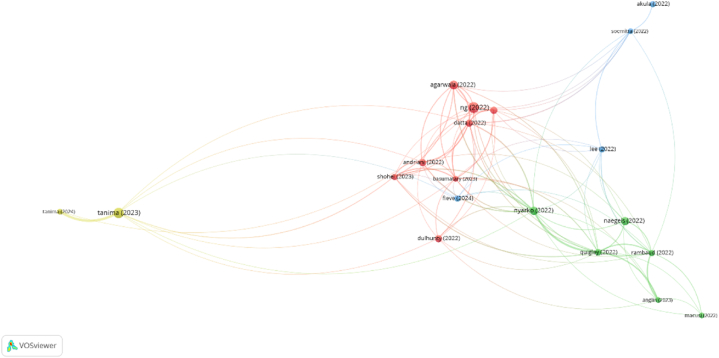
Bibliometric coupling analysis (1/1/2022-15/4/2024).

The identification of the most relevant research trends about the role of microfinance on women's empowerment has considered 158 articles about the topic, published between 1/1/2022 and 15/4/2024, following the same search and selection strategy that had been used in previous sections in order to carry out the productivity and mapping analyses. Amongst this literature, Fig. 6 represents the articles which have been cited at least 5 times, revealing the existence of four clusters which identify the hottest research trends.

The red cluster is the most populated one, and it also includes the most cited paper. The articles included mainly deal with the potential of microfinance to promote the empowerment of women, which is strongly related by these authors with the design of the specific microfinance program. So, the adequate tailoring of microfinance programs and actions appears as a first hot topic, catching the attention of a relevant number of academics. The majority of the papers included in this first group have studied the impacts of different programs that have taken place in India and Bangladesh.

The study of [220] sheds light on the dynamics of women empowerment in collective societies, where the individual empowerment of an entrepreneur woman has an impact on the entire population. This way, through adequately designed microfinance programs, women can play a role as active agents of change, whose impact can reach far beyond their own families, benefiting society as a whole [221]. Women empowerment can take place in different dimensions (social, economic, political and psychological) [222], and is strongly associated with the generation of social capital [223].

Nevertheless, though microfinance institutions can play a major role in empowering women in these different aspects, as [224,225] state, understanding the details of microcredit programs’ tailoring is critical in order to achieve the goal of empowering women: an inadequately developed microcredit program will not reinforce the role of women in society, but could be promoting women just rhetorically [226], giving men control over the financial resources borrowed by women, or even widening gender inequality [227].

The green cluster gathers six papers, mainly related with environmental conditions that can increase the potential of positive impact of microfinance programs, which are specially associated with cultural treats, which could be considered a second major trend in the study of the role of microfinance in the empowerment of women.

The study of [228] shows that women appear as better candidates for microloans, as they are better at reimbursing credits, so they should be the natural target of microfinance institutions. In spite of this, the opposite situation, where women often lack voice and power to protect their rights [229], is unfortunately frequent, even making women refrain from actions like religious expression, which will specially reduce their chance of getting funding from crowdfunding microfinance institutions [230]. [231]found out that the reasons for discouraging women to apply for financing when trying to be entrepreneurs are related with social norms and context. Following [232], positive gender empowerment norms and the presence of international founders enhance the outreach of microfinance institutions to empower women, while the egalitarian culture of a country influences the amount of funding achieved by women [233].

The blue cluster includes four papers which deal with the complementary knowledge-based resources needed to squeeze all the opportunities that may stem from a microfinance program. Therefore, the role of knowledge and training in the relationship between microfinance and women empowerment is another research stream that is currently attracting the attention of the academic community.

According to Ref. [234], financial literacy plays a major role in the relationship between women's empowerment and green microfinance. The knowledge base needed to profit from microfinance programs can also come from prior work experience [235]. [236] show that credit cooperative lending groups can significantly contribute to women entrepreneurship, providing their members with training and capacity building opportunities to improve their skills for sustainable businesses. In the context of Micro Waqf Bank [237], found out that mentoring can provide the knowledge needed to push the growth of a female micro-enterprise business.

Finally, the yellow cluster includes just two highly cited papers [238,239], that support the use of a critical dialogic accountability approach in order to analyze the actions and programs of microfinance institutions from a social justice perspective, putting the goals of the potential perceivers of microloans on the focus. More than setting a research trend, the authors propose a new analytical framework that has caught the attention of a relevant part of the academic community.

## 4 Discussion and conclusions

This study highlights the contribution of microfinance to women's empowerment, focusing on the benefits and drawbacks of microfinance in fighting poverty, gender-based violence and inequality of opportunities. Analyzing the relationship between microfinance and women's empowerment is critical for the decision-making of microfinance institutions, the design of social policies and the promotion of sustainable development of emerging sectors.

Women should have a major role in society. Public programs and private efforts to lead them into successful businesses, such as microfinance-backed entrepreneurship, can be a very powerful tool to promote economic and social growth. Without empowering women through gender equality in employment and education and reducing domestic abuse, meaningful social and economic transformation will be almost impossible to achieve. Traditional gender norms and practices should be reconsidered, leading to positive socio-cultural changes. Creating new opportunities for women to participate in entrepreneurial activities will help to emancipate them from poverty and social discrimination.

Consequently, several researchers have developed a vast body of academic literature to clarify social and economic challenges in achieving gender equality through microfinance. Filling a gap in the academic literature, this paper disentangles the knowledge base of the studies dealing with microfinance and women empowerment, presenting an overview and synthesis of the research on this topic, assessing its cognitive structure, evolution and trends using the Web of Science database and the VOSviewer software.

Since the first contribution to this research field, in 1998, there has been a remarkable boom in the academic literature, thanks to the contributions coming from 67 countries and 963 authors, published by 269 journals included in 36 research areas. Scientific production in this field showed limited growth in the early years (1998–2009, pioneering works), giving rise to some of the central papers in this field in an intermediate stage (2010–2015, laying the foundations), and generating a significant increase in recent years (2016–2021, consolidation and scientific development). This development can be associated with the relatively recent social transformations which are taking place all over the world, including the raise of gender equality, which has been fostered by initiatives of international organizations, especially the United Nations (UN). Thus, this international concern has caught the attention of researchers, who have developed studies useful for achieving the Sustainable Development Goals (SDGs), especially SDG#5, which involves gender equality and empowerment of all women and girls.

In addition, the focus on poverty (SDG#1) and inequalities (SDG#10) reduction has fostered research related with the impact of microfinance on women's empowerment. These studies suggest that providing microfinance services and promoting women's social inclusion could contribute significantly to reducing poverty and inequality of opportunities in emerging economies, granting the access of men and women to economic resources and microfinance services. However, events such as the COVID-19 pandemic caused a step back in poverty reduction (2020–2022) in developing countries that have not recovered yet: global inequality had its tipping point in 2020.

The papers that have shown a greater influence in the intellectual structure of this topic have been cited more than 140 times, with an average of more than 10 citations per year. These studies examine microfinance initiatives from different perspectives, including their impact on women's empowerment, socio-economic indicators, and business advocacy.

The countries that have contributed most to the scientific dissemination of this topic are the United States, with 130 publications, the United Kingdom, with 69 studies, and India, with 64 articles. The USA is a nation that has long contributed to the global advancement of the first microfinance revolution and the financial inclusion agenda, so its dominance is indicative of its commitment to research on this topic. Meanwhile, the UK has been working for years to eradicate poverty and promote gender equality, the latter being one of the four priorities of the 2022 UK Government Strategy for International Development. Finally, in India, Self-Help Groups (SHGs) dominate the microfinance sector as they have effectively provided financial services to the poor and advanced gender equality.

Similarly, the most prolific authors are Nadine Murshid, Isabelle Guerin, and Abdullah Al Mamun, with eight, seven and six publications, respectively. Their publications explore the impact of access to microcredit services on women's lives. Their findings demonstrate the researchers' commitment to this area of study.

The bibliometric mapping author analysis has visualized the networks of authors, finding four lines of research: Poverty alleviation, credit to empowerment, financial inclusion policies, and microcredit outcomes. Meanwhile, the bibliometric mapping keyword analysis has provided insight into the different areas that shape the intellectual structure of studying women's economic and social development and microfinance. The co-occurrence keyword analysis revealed four themes associated with this intellectual structure: poverty reduction, gender issues in entrepreneurship, microfinance and women empowerment, and women in microcredit.

## 5 Limitations and future lines of research

This study of the intellectual basis of the impact of microfinance on women's empowerment provides details on author networks, journals and study topics that can guide researchers interested in learning about this topic. However, this study has some limitations. These include the choice of the Web of Science, database rather than other recognized ones such as Scopus. Similarly, only one type of document, articles, was used for this research. Both restrictions could lead to missing some relevant publications, such as books. The final screening of publications may fall in some degree of subjectivity. Nevertheless, the use of PRISMA framework grants the maximum rigor in the selection process.

The study of the academic literature suggests new lines of research. For example, Peer-to-peer (P2P) lending platforms have emerged as a new method of obtaining loans in recent years. As a result, these kinds of organizations—like the Indonesian Amartha—have joined forces to support women's empowerment. Future studies might thus examine the effects of having access to this kind of business on women's economic and social advancement and the impact on society. Similarly, many SHG programs have modified Gender-Sensitive Indicators metrics to determine whether they have met their gender equity goals. Future research may analyze whether these programs successfully achieved their goals and provide fresh ideas for metrics to measure their results. In this way, policymakers could change or establish new mechanisms and public policies in partnership with microfinance institutions to support women's empowerment wholistically Bibliographic coupling has revealed the hottest topics of the research dealing with microfinance and women's empowerment, which are more than likely the most immediate paths that will be followed by the academic community. These topics are the importance of the tailoring of microfinance programs to fully profit from their potential; the impact of the environment, mainly the national culture, on the relationship between microfinance and women's empowerment; the importance of knowledge resources to squeeze the empowerment opportunities stemming from microfinance programs; and the use of a social justice perspective to analyze microcredits from the perceivers' point of view. Additionally, the current topics and trends identified in the study determine a necessary perspective for future research on identifying opportunities and challenges of green microfinance (especially microfinance programs and services) in empowering women in the context of climate change adaptation and mitigation.

**Fig. 7:**
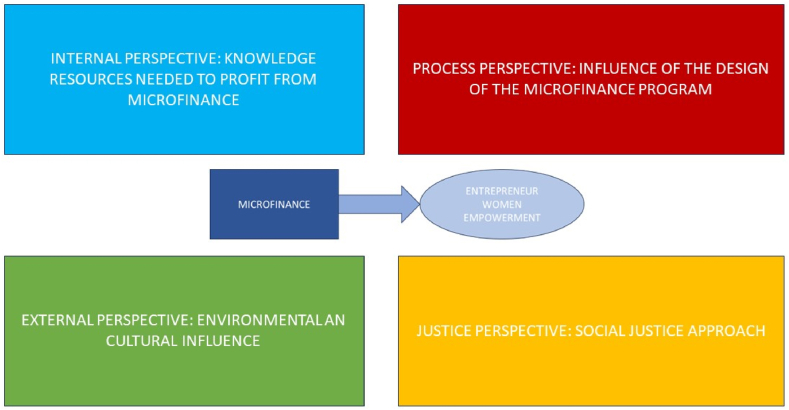
A four perspectives approach to the relationship between microfinance and women's empowerment.

These research streams can be associated with four different perspectives, mainly linked with four stakeholders, when analyzing the relationship between microfinance and women empowerment (Fig. 7): a process perspective, which deals with the design of the microfinance vehicle, which main responsibility lays on MFIs; an external perspective, associated with the environmental and cultural influence, linked with the entire society; an internal perspective, which consider the knowledge needed to profit from microfinance, directly related with the perceiver of the loan; and a justice perspective, which tries to grant microfinance programs fully oriented to the maximum welfare of the perceivers, which should be granted by policy makers.

The analysis of years of microcredits perceived by women in many countries has led the academic community to a relevant conclusion: microfinance can push women's empowerment, but does not always do it. The stakeholders involved in these programs, the MFIs, the society, the perceivers and the governments, can help to make the relationship work, so that microloans can truly help to foster the empowerment of women, helping to build a fairer world not just for them, but for the entire society.

## CRediT authorship contribution statement

**Jenny Maldonado-Castro:** Writing – original draft, Validation, Software, Resources, Methodology, Investigation, Formal analysis, Data curation, Conceptualization. **Rocío Gallego-Losada:** Writing – review & editing, Writing – original draft, Visualization, Validation, Supervision, Project administration, Methodology, Investigation, Formal analysis, Data curation, Conceptualization. **Antonio Montero-Navarro:** Writing – review & editing, Writing – original draft, Visualization, Validation, Supervision, Project administration, Methodology, Investigation, Formal analysis, Data curation, Conceptualization.

## Ethics statement

Review and/or approval by an ethics committee was not needed for this study because the research did not involve experimentation on animals or include human subjects.

Informed consent was not required for this study because the research did not involve experimentation on animals or include human subjects.

## Data availability

According with the nature of this work, no data associated with this study has been deposited into a publicly available repository. If necessary, data will be made available on request.

## Declaration of competing interest

The authors declare that they have no known competing financial interests or personal relationships that could have appeared to influence the work reported in this paper.
